# Communicating about microbicides with women in mind: tailoring messages for specific audiences

**DOI:** 10.7448/IAS.17.3.19151

**Published:** 2014-09-08

**Authors:** Sekou Sidibe, Allison P Pack, Elizabeth E Tolley, Elizabeth Ryan, Caroline Mackenzie, Emily Bockh, George Githuka

**Affiliations:** 1 Social & Behavioural Health Sciences, FHI360, Durham, NC, USA; 2 Social Marketing and Communication, FHI 360, Washington, DC, USA; 3 Research, FHI 360, Nairobi, Kenya; 4 National AIDS and STI Control Programme, Nairobi, Kenya

**Keywords:** microbicide, communication, women, HIV prevention, messages, Kenya

## Abstract

**Introduction:**

Current HIV prevention options are unrealistic for most women; however, HIV prevention research has made important strides, including on-going development of antiretroviral-based vaginal microbicide gels. Nevertheless, social-behavioural research suggests that women's ability to access and use new HIV prevention technologies will be strongly influenced by a range of socio-cultural, gender and structural factors which should be addressed by communications and marketing strategies, so that these products can be positioned in ways that women can use them.

**Methods:**

Based on an extensive literature review and in-country policy consultation, consisting of approximately 43 stakeholders, we describe barriers and facilitators to HIV prevention, including potential microbicide use, for four priority audiences of Kenyan women (female sex workers [FSWs], women in stable and discordant relationships, and sexually active single young women). We then describe how messages that position microbicides might be tailored for each audience of women.

**Results:**

We reviewed 103 peer-reviewed articles and reports. In Kenya, structural factors and gender inequality greatly influence HIV prevention for women. HIV risk perception and the ability to consistently use condoms and other prevention products often vary by partner type. Women in stable relationships find condom use challenging because they connote a lack of trust. However, women in other contexts are often able to negotiate condom use, though they may face challenges with consistent use. These women include FSWs who regularly use condoms with their casual clients, young women in the initial stages of a sexual relationship and discordant couples. Thus, we consider two approaches to framing messages aimed at increasing general awareness of microbicides – messages that focus strictly on HIV prevention and ones that focus on other benefits of microbicides such as increased pleasure, intimacy or sexual empowerment, in addition to HIV prevention.

**Conclusions:**

If carefully tailored, microbicide communication materials may facilitate product use by women who do not currently use any HIV prevention method. Conversely, message tailoring for women with high-risk perception will help ensure that microbicides are used as additional protection, together with condoms.

## Introduction

In Kenya, HIV prevalence has fallen from 7.2% in 2007 to 5.6% in 2012 among adults aged 15–64 [1, 2]. However, women continue to be disproportionally affected by HIV; prevalence rates were 6.9% among women, compared to 4.4% among men [1]. In addition, approximately 40% of new infections have occurred within stable couples [1]. Women's need for new HIV prevention technologies remains a priority.

Recent trials have demonstrated the effectiveness of new antiretroviral (ARV)-based HIV prevention products. Oral pre-exposure prophylactic (PrEP) use of tenofovir or Truvada (tenofovir and emtricitibine) was shown to reduce HIV transmission in heterosexual discordant couples (by 62% for tenofovir-only and 73% for Truvada) [3–5]. Additionally, the CAPRISA 004 clinical trial in South Africa produced a proof of concept for peri-coital vaginal use of 1% tenofovir gel in reducing HIV transmission to heterosexual women [6].

While oral PrEP has been approved for use in high-risk populations in the United States [7], decisions about vaginal microbicide gel may still be several years away as regulators await confirmatory results from the FACTS 001 trial. Furthermore, despite commonalities between oral and vaginal ARV-based products for HIV prevention, their introduction strategies may require different approaches.

## The need for tailored introduction strategies

Social-behaviour research conducted alongside microbicide clinical trials suggests women's acceptability of new HIV prevention technologies, and their ability to access and use them, will be strongly influenced by the way products are positioned by communication and marketing strategies [8]. For messages to be effective, they must be tailored; different audiences will perceive different sources of risk, outcome expectations and barriers to action. For example, previous research suggests women's ability to identify their HIV risk varies considerably by geographic and sexual relationship context [9–12]. Research globally has associated low rates of condom use, especially among women in stable relationships, with the perception that condoms are primarily for HIV prevention. And, because HIV is associated with high-risk sexual behaviour, the request for condom use within a stable relationship connotes unfaithfulness. Female condom programmes have worked to reverse such connotations by emphasizing the use of female condoms for contraception and by promoting their use in loving relationships [13]. It will be equally important to develop and test potential ways to position microbicide use to avoid stigmatization.

Some researchers and advocates have suggested that vaginal microbicide gel might be less stigmatized if it is positioned as a sexual lubricant [14]. However, although the notion that gel use increases sexual pleasure could be positively perceived by some, it may be negatively perceived by others. Similarly, if gel is promoted for use among audiences in which condom use is a norm, communication strategies should model dual use of gel and condoms to avoid the substitution of more efficacious condoms with less efficacious microbicide products. Therefore, introduction strategies, including the content and framing of messages, will need to be tailored to various audiences if new technologies are to be widely accessed and used.

*Communicating about Microbicides with Women in Mind* is a three-phase project to assist Kenyan policymakers, programme implementers and advocacy groups in planning for future introduction of ARV-based vaginal microbicide gel, once proven effective. Specifically, the project aims to develop a suite of communication materials tailored to the needs of potential users and to assess the effects of message framing on interest in microbicide use, negative attitudes towards microbicides and potential condom migration.

In this paper, we draw on an extensive literature review and in-country policy consultation, conducted during the first project phase, to describe four potential audiences who might benefit from microbicide use. We also discuss how communication materials might require tailoring for each audience.

## Methods

### Literature search strategy

During the first phase, we systematically searched peer-reviewed articles, from January to July 2012, using PubMed and Google Scholar. We sought peer-reviewed articles of academic quality that identified barriers and facilitators to HIV prevention among four potential end-user audiences representing different sexual relationship contexts: female sex workers (FSWs), women in stable relationships, women in discordant relationships, and sexually active, single young women. These priority audiences were selected based on their high HIV prevalence rates and previous research of key populations who might benefit from vaginal microbicide use [6, 9, 10]. Other data sources included national surveillance system data reports, such as the AIDS indicator surveys and demographic health surveys.

The search included medical subject headings (MeSH) terms for HIV and AIDS, terms associated with each of the four priority audiences (“sex work,” “FSWs,” “couples,” “serodiscordant couples,” “young women,” “women,” “youth,” and “adolescents”), and key terms associated with HIV prevention (“HIV testing,” “condoms,” “microbicides,” “PrEP,” “sexual relationships,” “communication,” “discussion,” “negotiation,” “HIV risk perception,” “sexual pleasure,” “sexual practices,” “sexual power,” “gender-based violence/coercion,” “stigma,” “education,” “social support,” “money,” “substance use,” and “alcohol use”).

### Inclusion and exclusion criteria

**Figure 1:**
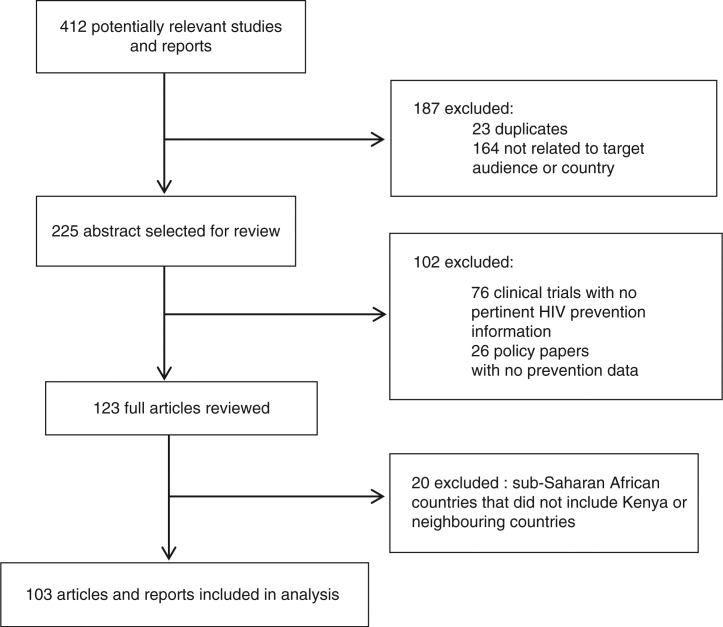
Flow chart of included articles and reports.

Studies of any design were eligible for inclusion if they addressed HIV prevention among one or more of the specified audiences and if they were published between 1990 and 2012. Articles were also included in the analysis if they reported findings from Kenya or neighbouring countries, including Uganda, Rwanda and Tanzania. Several multi-site studies in sub-Saharan African were also included when Kenya was one of the prioritized countries. All surveillance reports specific to Kenya were included in the analysis. Articles and reports were excluded if they did not focus on any of the priority audiences, HIV prevention, or Kenya and its neighbouring countries (Figure 1).

### Analysis

The search yielded 103 unique articles and reports (Table 1), 96 of which were specific to Kenya; the remaining pertained to neighbouring sub-Saharan African countries. Several articles (*n*=27) included information on men and young boys; they were retained because they contained information relevant to the priority audiences.

**Table 1:** Geographical focus of the articles

Geographical focus	Sex workers	Young women	Stable relationships	Discordant couples	Other groups	Total (%)
Kenya	38	25	7	5	21	96 (93.20)
Sub-Saharan Africa	2	0	0	0	5	7 (6.79)
Total	40	25	7	5	27	103 (100)

Articles and reports were uploaded into NVivo 9.2, read for content and coded to identify 26 broad themes, including risk perception, sexual practices, communication and condom use as well as priority audiences. Articles and reports were categorized and analysed by audiences based on specific themes; those containing data on multiple audiences were coded for each relevant audience. Data pertaining to multiple audiences or themes were also double coded. For each main theme, we created an Excel matrix organized by article, with up to three illustrative excerpts across the columns, and colour-coded by audience group. These thematic matrices helped identify similarities and differences among audiences in terms of HIV prevention needs and challenges.

Data were presented and discussed during a two-day national policy consultation, conducted in Naivasha, Kenya, in collaboration with the National AIDS and STI Control Programme and the Kenya Medical Research Institute. Participants included 43 stakeholders comprising Kenyan policymakers, programme managers and civil society advocates, as well as representatives from international non-governmental organizations (NGOs) and funding agencies. The potential introduction of ARV-based HIV prevention in Kenya, including various priority audiences for microbicide communication materials, was discussed.

## Results

### Literature review

The varying social contexts within which women and their partners live, work and love create unique challenges for HIV prevention efforts (Table 2). The number and type of partners, in large part influenced by structural factors and gender inequality [15], affect women's HIV risk perception and their ability to respond to that risk [16]. For example, the ability to use condoms consistently and correctly often varies by partner type [9, 17, 18]. As Kenya considers microbicide introduction, understanding current HIV prevention behaviour and related sexual practices in Kenya may prove useful in anticipating potential barriers and facilitators to new product use for various audiences.

**Table 2:** Focused, summary results from the literature review by audience

Female sex workers	Women in stable relationships
Partner information • Partners include clients, regular partners, boyfriends, husbands – of unknown status • Low decision-making power with regular partners • Sex is often planned • Potential for violence HIV risk perception and information source • High-risk perception • Receive HIV information from peer educators Condom use • High condom use with clients • Low condom use with regular partners Microbicide considerations • Lubrication could facilitate condom use • Some perceive that clients prefer dry sex • Multiple partners, substance use might affect adherence • Vaginal washing might reduce effectiveness	Partner information • Some have one regular partner of unknown status; others have additional partners of unknown status • Low decision-making power in general • Potential for violence HIV risk perception and information source • Low-risk perception • Receive HIV information and testing in antenatal care settings Condom use • Some condom use at start of relationship • Low/no condom use once committed Microbicide considerations • Partner involvement shown to improve adherence • High acceptability among married trial participants • Some concerns about stigma; may imply infidelity
**Discordant couples**	**Single young women (15 to 24 years)**

Partner information • Some have one regular partner only; others have additional partners of unknown status • Low decision-making power with additional partners • Potential for violence HIV risk perception and information source • Low-risk perception prior to testing and disclosure, but high-risk perception after testing, disclosure • Receive HIV information from health system Condom use • High condom use with negative partner, if relationship continues • Low condom use with additional partners Microbicide considerations • Could protect discordant couples who wish to conceive • May be useful for HIV-negative women before HIV-positive partner initiates treatment	Partner information • Partners can include transactional, cross-generational sex partners, regular partners, boyfriends – of unknown status • Low decision-making power in general • Sex is not often planned • Potential for violence HIV risk perception and information source • Low-risk perception • Receive HIV information from schools and youth-friendly clinics and peers, but information is limited Condom use • Some condom use at start of intimate relationship • Low condom use in general Microbicide considerations • Interest in new, effective products • Concern about use disclosure • Some concern about product insertion, adherence

#### Female sex workers

The 2012 Most-at-Risk Populations (MARPs) Surveillance Report estimates that the HIV prevalence rate among FSWs in Kenya is between 24 and 50% [19]. Several Kenyan studies [19–24] suggest that FSWs work within a culture of violence, commonly triggered by substance use and discussions of condom use and payment. The illegal nature of sex work perpetuates discrimination, deterring FSWs from reporting the violence or seeking assistance from law enforcement [20–21]. Moreover, economic necessity negatively affects FSWs’ ability to negotiate condom use; inconsistent use often results from a client's willingness to pay more for unprotected sex [9, 25].

Nevertheless, HIV risk perception is high among many sex workers [16, 25], and many are able to respond to their risk. FSWs report considerable awareness of STI symptoms, high rates of treatment-seeking behaviours, HIV testing and subsequent knowledge of HIV status [9, 18]. A number of studies conducted across Kenya report high rates of consistent condom use among FSWs [9, 24, 26].

Yet, condom use among FSWs is strongly associated with partner type [9, 16, 18, 27, 28]; higher rates of condom use are reported with casual partners or clients than with regular or primary partners. Some studies suggest that for FSWs, primary partners are considered less risky than casual partners [23, 28], a theme which is consistent with women in other contexts.

One study assessing vaginal microbicide gel acceptability among FSWs revealed that gel could be promoted as a lubricant to facilitate condom use, thus enhancing protection [10]. However, a study assessing intravaginal practices and implications for new HIV prevention products among FSWs in urban Kenya revealed that vaginal washing practices, often influenced by perceived or real male preference for “dry sex,” and anal sex were also common [28, 29]. Such practices may reduce the effectiveness of vaginal microbicides.

#### Women in stable relationships

According to the 2012 KAIS, HIV prevalence among women in stable relationships was estimated to be 5.3% [1]. Moreover, an estimated 44% of new infections are thought to occur within stable relationships, according to the 2009 Kenya HIV Response and Modes of Transmission Analysis [30]. Studies suggest that women in stable relationships perceive their risk for HIV to be low [22, 31] and are therefore rarely tested for HIV outside of antenatal contexts [32]; as part of the 2007 KAIS, most women (about 60%) reported being tested within the context of antenatal services [2].

Within stable relationships, men and women report different patterns of sexual behaviour. While women report approximately two lifetime partners, men report more than seven. Similarly, concurrent relationships were reported by 1% of women in monogamous relationships and 2.6% of women in polygamous relationships, compared to 7 and 10% of men [2].

Kenyan men are important decision makers in sexual matters, from decisions about the number and timing of children to the use of contraceptive and disease prevention methods. In contrast, women have been socialized to comply with their partner's desires for sex and pleasure [32].

Women in stable relationships face barriers to requesting condom use, largely because such requests raise suspicions of unfaithfulness and increase the potential for violence [33–36]. And, while condom use for contraception appears more acceptable than for disease prevention, less than 2% of women in stable relationships report using condoms for contraception [37]. Cross-sectional studies across Kenya report low spousal communication related to HIV prevention, further suggesting that women in stable relationships have little ability to negotiate condom use or other safe sex practices [35–37].

#### Discordant couples

According to the KAIS 2012, there are an estimated 260,000 HIV-discordant couples in Kenya [1]. Among couples in which both partners received HIV test results, 5% were identified as HIV-discordant and 3% were concordant HIV-positive [1]. Yet, risk perception is generally low for these couples before testing. Indeed, 53% of men and women who were tested as part of the 2012 KAIS and found to be in HIV-discordant relationships were unaware of their status [1]. Similarly, a study conducted in Mombasa revealed that women were unaware that their primary partners were involved in other relationships [38].

Before diagnosis, women and men in discordant relationships are like other stable couples in which women's fidelity and fertility are highly valued. In fact, fertility continues to be important even within HIV-affected relationships. The 2007 KAIS reported that about one-fourth of women who self-declared as HIV positive reported wanting a/another child [2], yet fear of stigma is thought to limit the use of necessary PMTCT services for HIV-positive women [39–41].

Fear of stigma has also been found to inhibit HIV status disclosure between partners, regardless of relationship length. However, once discordancy has been disclosed, if couples choose to remain together, both HIV risk perception and the ability to negotiate risk reduction behaviours can increase with effective HIV counselling [11].

#### Single young women

An estimated 5.6% of sexually active young women aged 15–24 were identified as HIV positive in the 2007 KAIS study [2]. Among young women who participated in the 2012 KIAS, 66% reported ever having sex [1]; other studies suggest that women initiate sex at young ages and that sexual activity often begins within the first month of a new relationship [12, 42].

Despite widespread reports of multiple concurrent partnerships among Kenyan youth [17, 43, 44], HIV risk perception and subsequent condom use is low for young women [45, 46]; only 11% reported consistent condom use in the 2012 KAIS [1]. As with women in other contexts, condom use varies by partner type; it primarily occurs at the start of a relationship when risk is perceived to be high [12, 43] and is abandoned once “trust” is established [43]. Young women's ability to negotiate condom use is also compromised by gender, age and economic disparities, especially in situations involving cross-generational and transactional sex [12, 46, 47]. While some use these exchanges to meet basic needs [43, 48, 49], others are motivated by the potential for supplementary gifts [46, 49].

Misinformation about HIV transmission is pervasive among many young women [17, 46–50]. Despite lessons on life-skills, teachers in Kisumu and Nakuru have reported feeling unprepared to talk about sexuality in the classroom, in part, due to parental concerns [51]. Similarly, cultural taboos about adolescent sexuality pose barriers to purchasing condoms, negotiating condom use with partners [48, 52], or visiting a health centre for HIV and STI testing. However, providing an increase in youth-friendly HIV testing and counselling centres in Kisumu resulted in an increase in HIV testing [44].

Montandon *et al*. qualitatively examined the potential acceptability and use of microbicides among young women in Kisumu [49] in 2008. This study suggested that the unplanned nature of adolescent sexual activity, a fear of vaginally inserting or trying new, “experimental” products, and concerns about product efficacy will likely impede vaginal microbicide use for young women [49].

#### Policy consultation

Results from the literature review, shared at the Naivasha consultation, provided context for participants to prioritize audiences they felt would be most suited for microbicide communication materials. With financial and human resources likely to be a challenge for new product implementation, some participants felt it would be important to prioritize known risk groups (FSWs and/or HIV-negative women in discordant couples) already identified as a national priority for HIV prevention.

However, participants also expressed concern about the effects of stigma, and whether focusing on known risk groups would inadvertently lead to the stigmatization and subsequent non-use of microbicides by other women. Some participants felt strongly that while women in stable relationships and young women may not typically be considered most-at-risk, current HIV prevention options are not realistic for them.

Consequently, a group of known high-risk women, (FSWs) and two groups of at-risk women who have not traditionally been targeted with HIV prevention information (single young women aged 15–24 and women in stable relationships) were identified as primary audiences for the *Communicating about Microbicides with Women in Mind* project. Men and health care providers were identified as secondary audiences.

Finally, meeting participants also emphasized that civil society organizations should work closely with the government to develop appropriate procedures and regulations for microbicide introduction. They agreed the government should drive the agenda for the introduction of ARV-based HIV prevention, including the development of national guidelines. A project advisory committee (PAC), consisting of key governmental and non-governmental stakeholders, was therefore established to guide the *Communicating about Microbicides with Women in Mind* project.

## Discussion

Social marketing and communication strategies can potentially increase the uptake of new HIV prevention technologies, including microbicides. UNAIDS has long acknowledged the role of social marketing as an effective tool in the global response to HIV/AIDS [53]. In a review of more than 6500 studies, PSI conducted an analysis of social marketing approaches and programmes, demonstrating the effectiveness of social marketing for changing health behaviours. Twenty studies demonstrated increases in HIV risk perception, knowledge, and self-efficacy; 18 demonstrated increases in condom use and HIV testing; and eight demonstrated reductions in HIV prevalence [54]. However, the design and implementation of effective strategies relies on a solid understanding of priority audiences and the many factors that influence their behaviours. This information determines the most effective way of positioning products to generate interest in use.

Because vaginal microbicides are likely to be partially effective, they may need to be promoted within the context of condom use. Experience from programmes that have promoted dual protection against pregnancy and HIV may be useful. Unfortunately, there have been few quality studies documenting the effectiveness of behavioural interventions for promoting dual protection. Yet, many organizations that have implemented dual protection interventions have documented lessons learned; these include the need to tailor messages to specific HIV contexts and the fact that a dual protection message is not always appropriate [55].

Microbicide introduction strategies will need to consider how to position microbicides so they:

1. Can be used by all types of sexually active women
2. Do not connote lack of trust or infidelity
3. Are used when condom use is not possible or as a back-up for condoms
4. Do not replace condoms when condom use is possible

To this end, it will be important to determine whether microbicide awareness-raising materials, should focus exclusively on HIV prevention or more prominently on other benefits such as sexual pleasure, intimacy, and/or female empowerment. Which type of framing generates the most interest in microbicides will likely differ depending on the target audience. At the same time, it will be important to avoid linking microbicides to stigmatizing behaviours such as unfaithfulness or sex work. And finally, because microbicides are currently not as effective as condoms, materials must avoid promoting them in a way that would result in decreased condom use among those who use condoms with all or some of their partners.

Widespread use of microbicides will require more than awareness, however; it will also require intensive counselling about risks, benefits, feasibility and correct use of microbicides. Educational materials, tailored for specific audiences, offer an opportunity for more detailed discussion about these topics and potential adherence challenges. Educational materials designed for FSWs and HIV-negative women in discordant relationships, for example, will need to emphasize the importance of combining microbicides with condom use, as opposed to substituting condom use with microbicides. As indicated by the literature, FSWs have high-risk perception and an ability to negotiate condom use with clients, yet they are also economically motivated and may view condom-less sex as an opportunity to obtain additional cash. Materials will need to deter this behaviour and promote dual use of microbicides and condoms as enhancing protection and potentially facilitating condom use.

Moreover, materials for FSWs will need to address various contexts in which microbicides may be used, including with primary partners. Because sex is often anticipated for these women, materials might want to encourage daily or routine use of microbicides. And finally, materials will need to address various sexual practices reportedly common among some FSWs, including vaginal douching and anal sex.

For young women and women in stable relationships, however, educational materials will need to acknowledge that partial protection from microbicides is better than no protection at all. Moreover, materials should provide detailed information on HIV risk and prevention options. As indicated by the literature, despite widespread reports of concurrent partnerships, many of these women do not perceive themselves to be at risk and have not used, or do not currently use, condoms with their partners. Likewise, with many young women and women in stable relationships reporting difficulty negotiating safe sex with their partners, educational materials should provide guidance to ensure women can decide whether and/or how they should involve their partners in discussions about microbicide use.

For young women, in particular, materials will need to address the fact that many of them have limited experiences with reproductive health services, may fear necessary HIV testing or the insertion of new vaginal products, and often do not plan for sex.

Limitations for this project include the fact that there are currently few studies available on microbicide acceptability in Kenya and HIV prevention among women in stable relationships and women in discordant couples. Moreover, without an available product, much of the work we have conducted is based on hypothetical assessments.

## Conclusions

Findings from this project address a critical gap in our current approach to HIV prevention communication by providing evidence-based recommendations that should be considered for communication materials in order to support the future introduction of vaginal microbicide gels in Kenya. We believe that these recommendations can be used to create effective materials and will contribute to increased use of microbicides by women in a variety of sexual contexts – including young women and those in stable relationships – who have not traditionally been considered at high risk of HIV, but may actually be some of the most-at-risk.
